# Bis(triphenyl­guanidinium) tetra­chlorido­cuprate(II)

**DOI:** 10.1107/S1600536808023404

**Published:** 2008-07-31

**Authors:** P. S. Pereira Silva, N. D. Martins, M. Ramos Silva, A. Matos Beja

**Affiliations:** a CEMDRX, Physics Department, University of Coimbra, P-3004-516 Coimbra, Portugal

## Abstract

The structure of the title compound, (C_19_H_18_N_3_)_2_[CuCl_4_], consists of square-planar [CuCl_4_]^2−^ anions and triphenyl­guanidinium cations. The Cu^II^ ion occupies a crystallographic inversion centre. In the cation, the dihedral angles between the phenyl rings and the plane defined by the central guanidinium fragment are in the range 51.9 (4)–64.4 (3)°. N—H⋯Cl hydrogen bonds assemble the ions into infinite chains running along the *b* axis.

## Related literature

For related literature, see: Bian *et al.* (2005 ▶); Kemme *et al.* (1988 ▶); Klement *et al.* (1995 ▶); Pereira Silva *et al.* (2006 ▶); Pereira Silva *et al.* (2007 ▶).
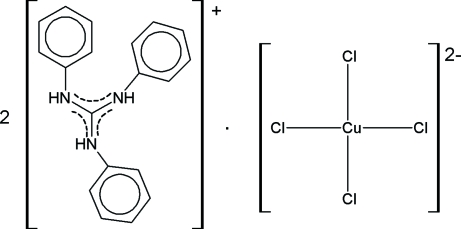


## Experimental

### 

#### Crystal data

(C_19_H_18_N_3_)_2_[CuCl_4_]
*M* *_r_* = 782.08
Monoclinic, 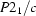

*a* = 11.5893 (13) Å
*b* = 8.2404 (9) Å
*c* = 22.364 (2) Å
β = 119.423 (7)°
*V* = 1860.3 (3) Å^3^
*Z* = 2
Mo *K*α radiation
μ = 0.91 mm^−1^
*T* = 293 (2) K
0.21 × 0.10 × 0.04 mm

#### Data collection

Bruker APEX2 CCD area-detector diffractometer
Absorption correction: multi-scan (*SADABS*; Sheldrick, 2003 ▶) *T*_min_ = 0.744, *T*_max_ = 0.964
27709 measured reflections
3304 independent reflections
1405 reflections with *I* > 2σ(*I*)
*R*_int_ = 0.164

#### Refinement

*R*[*F* ^2^ > 2σ(*F* ^2^)] = 0.097
*wR*(*F* ^2^) = 0.319
*S* = 1.05
3304 reflections
223 parameters
H-atom parameters constrained
Δρ_max_ = 1.28 e Å^−3^
Δρ_min_ = −0.52 e Å^−3^

### 

Data collection: *APEX2* (Bruker, 2005 ▶); cell refinement: *SAINT* (Bruker, 2003 ▶); data reduction: *SAINT*; program(s) used to solve structure: *SHELXS97* (Sheldrick, 2008 ▶); program(s) used to refine structure: *SHELXL97* (Sheldrick, 2008 ▶); molecular graphics: *PLATON* (Spek, 2003 ▶); software used to prepare material for publication: *SHELXL97*.

## Supplementary Material

Crystal structure: contains datablocks global, I. DOI: 10.1107/S1600536808023404/er2054sup1.cif

Structure factors: contains datablocks I. DOI: 10.1107/S1600536808023404/er2054Isup2.hkl

Additional supplementary materials:  crystallographic information; 3D view; checkCIF report

## Figures and Tables

**Table 1:** Hydrogen-bond geometry (Å, °)

*D*—H⋯*A*	*D*—H	H⋯*A*	*D*⋯*A*	*D*—H⋯*A*
N1—H1⋯Cl2^i^	0.86	2.28	3.126 (8)	167
N2—H2⋯Cl1^ii^	0.86	2.51	3.218 (8)	140
N3—H3⋯Cl1	0.86	2.44	3.253 (9)	159

Symmetry codes: (i) 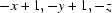; (ii) 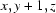.

**Fig. 1.**
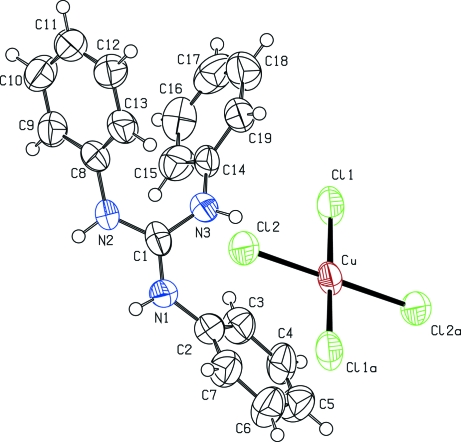
ORTEPII (Spek,2003) plot of the title compound. Displacement ellipsoids are drawn at the 50% probability level.

**Fig. 2.**
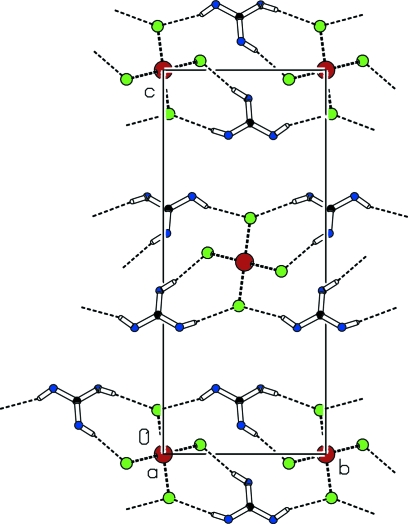
Packing diagram, viewed down the a axis, with the hydrogen bonds depicted as dashed lines. The phenyl rings have been omitted for clarity.

## supplementary crystallographic information

### Comment

Molecular based magnets, systems in which molecular orbitals are crucial in mediating the magnetic interaction, are often synthesized by mild-chemistry conditions. Furthermore, the molecules/ions often assemble in low dimensional compounds providing easier systems to study both theoretical and experimentally. Some systems are even classified as Single Molecule Magnets, since the organically bridged metal clusters, exhibit magnetic properties similar to those observed in conventional bulk magnets like remanence and hysteresis (Bian *et al.*, 2005). The title compound, (I), Fig.1, was synthesized within a project aiming at developing new molecular based magnets. Compound (I) is built up from triphenylguanidinium cations and CuCl_4_^2-^ anions. The CN_3_ fragment of the guanidinium group in (I) is planar, as expected for *sp*^2^ hybridization of the central C atom. The bond lengths C1—N1 [1.324 (12) Å], C1—N2 [1.332 (12) Å] and C1—N3 [1.347 (12) Å] are within the range expected for a delocalized C-N bond. The dihedral angles between the ring planes and the plane defined by the central guanidinium fragment are 51.9 (4)°(C2—C7), 59.8 (4)°(C8—C13) and 64.4 (3)°(C14—C19). The corresponding angles for other triphenylguanidinium salts reported in the literature are within the range 32.6 (3)–70.2 (3)° (Kemme *et al.*, 1988; Klement *et al.*, 1995; Pereira Silva *et al.*, 2006, 2007). This variability attests the flexibility of the triphenylguanidinium cation. The Cu^II^ ion occupies a crystallographic inversion centre and the environment around the metal ion is square-planar. There are hydrogen bonds between all the NH groups and the Cl^-^ ions, each CuCl_4_^2-^ anion being linked to four cations, forming infinite chains along the [010] direction (Fig. 2, Table 2).

### Experimental

Copper(II) chloride dihydrate (Riedel-de-Haën, pro analysis >99%, 0.125 mmol) was dissolved in 50 ml of hot water and triphenylguanidine (TCI, 97%, 0.25 mmol) was dissolved in ethanol (50 ml). The two solutions were mixed and several drops of HCl (Merck, 37%) were added. The solution was left to evaporate at room temperature and pressure. Green single crystals of (I) were obtained from the solution after a few days.

### Refinement

Several crystals of (I) were probed but the *R*_int_ of all data collections was relatively high, reflecting the poor quality of the crystals. The maximum peak in the final difference Fourier map is 1.29 e Å^-3^ situated at 1.14 Å from the atom Cl1 and at 1.17 Å from the heavier metal atom. H atoms were placed at calculated positions and refined as riding on their parent atoms, using *SHELXL97* (Sheldrick, 2008) defaults [C—H = 0.93 Å, N—H = 0.86 Å and *U*_iso_(H) = 1.2*U*_eq_(C,*N*).

### Crystal data

**Table:**

(C_19_H_18_N_3_)_2_[CuCl_4_]	*F*_000_ = 806
*M**_r_* = 782.08	*D*_x_ = 1.396 Mg m^−^^3^
Monoclinic, *P*2_1_/*c*	Mo *K*α radiation λ = 0.71073 Å
Hall symbol: -P 2ybc	Cell parameters from 1887 reflections
*a* = 11.5893 (13) Å	θ = 2.7–15.3º
*b* = 8.2404 (9) Å	µ = 0.91 mm^−^^1^
*c* = 22.364 (2) Å	*T* = 293 (2) K
β = 119.423 (7)º	Prism, green
*V* = 1860.3 (3) Å^3^	0.21 × 0.10 × 0.04 mm
*Z* = 2	

### Data collection

**Table:**

Bruker APEX2 CCD area-detector diffractometer	3304 independent reflections
Radiation source: fine-focus sealed tube	1405 reflections with *I* > 2σ(*I*)
Monochromator: graphite	*R*_int_ = 0.164
*T* = 293(2) K	θ_max_ = 25.1º
φ and ω scans	θ_min_ = 2.0º
Absorption correction: multi-scan(SADABS; Sheldrick, 2003)	*h* = −13→13
*T*_min_ = 0.744, *T*_max_ = 0.964	*k* = −9→9
27709 measured reflections	*l* = −26→26

### Refinement

**Table:**

Refinement on *F*^2^	Secondary atom site location: difference Fourier map
Least-squares matrix: full	Hydrogen site location: inferred from neighbouring sites
*R*[*F*^2^ > 2σ(*F*^2^)] = 0.097	H-atom parameters constrained
*wR*(*F*^2^) = 0.319	*w* = 1/[σ^2^(*F*_o_^2^) + (0.1615*P*)^2^ + 0.2636*P*] where *P* = (*F*_o_^2^ + 2*F*_c_^2^)/3
*S* = 1.05	(Δ/σ)_max_ < 0.001
3304 reflections	Δρ_max_ = 1.29 e Å^−^^3^
223 parameters	Δρ_min_ = −0.52 e Å^−^^3^
Primary atom site location: structure-invariant direct methods	Extinction correction: none

### Special details

**Table:**

Geometry. All e.s.d.'s (except the e.s.d. in the dihedral angle between two l.s. planes) are estimated using the full covariance matrix. The cell e.s.d.'s are taken into account individually in the estimation of e.s.d.'s in distances, angles and torsion angles; correlations between e.s.d.'s in cell parameters are only used when they are defined by crystal symmetry. An approximate (isotropic) treatment of cell e.s.d.'s is used for estimating e.s.d.'s involving l.s. planes.
Refinement. Refinement of *F*^2^ against ALL reflections. The weighted *R*-factor *wR* and goodness of fit *S* are based on *F*^2^, conventional *R*-factors *R* are based on *F*, with *F* set to zero for negative *F*^2^. The threshold expression of *F*^2^ > σ(*F*^2^) is used only for calculating *R*-factors(gt) *etc*. and is not relevant to the choice of reflections for refinement. *R*-factors based on *F*^2^ are statistically about twice as large as those based on *F*, and *R*- factors based on ALL data will be even larger.

### Fractional atomic coordinates and isotropic or equivalent isotropic displacement parameters (Å^2^)

**Table:**

	*x*	*y*	*z*	*U*_iso_*/*U*_eq_	
Cu	0.5000	0.0000	0.0000	0.0567 (7)	
Cl1	0.6357 (3)	−0.0354 (3)	0.11412 (14)	0.0719 (9)	
Cl2	0.4205 (3)	0.2267 (4)	0.02411 (15)	0.0826 (11)	
N1	0.7105 (8)	0.4791 (9)	0.0748 (4)	0.055 (2)	
H1	0.6701	0.5646	0.0521	0.066*	
N2	0.7200 (9)	0.5995 (10)	0.1697 (4)	0.062 (2)	
H2	0.7380	0.6909	0.1576	0.075*	
N3	0.7583 (8)	0.3232 (10)	0.1706 (4)	0.057 (2)	
H3	0.7238	0.2389	0.1453	0.069*	
C1	0.7289 (10)	0.4673 (11)	0.1380 (6)	0.055 (3)	
C2	0.7497 (12)	0.3671 (13)	0.0409 (6)	0.060 (3)	
C3	0.8688 (11)	0.2853 (14)	0.0731 (6)	0.069 (3)	
H3A	0.9255	0.2991	0.1199	0.083*	
C4	0.9037 (14)	0.1834 (15)	0.0360 (7)	0.085 (4)	
H4	0.9849	0.1303	0.0577	0.102*	
C5	0.8197 (15)	0.1595 (17)	−0.0327 (8)	0.084 (4)	
H5	0.8431	0.0888	−0.0574	0.101*	
C6	0.7024 (15)	0.2395 (16)	−0.0646 (6)	0.086 (4)	
H6	0.6462	0.2265	−0.1115	0.103*	
C7	0.6666 (12)	0.3400 (14)	−0.0272 (6)	0.074 (3)	
H7	0.5842	0.3903	−0.0489	0.089*	
C8	0.6834 (11)	0.6040 (13)	0.2223 (5)	0.059 (3)	
C9	0.7498 (11)	0.7120 (13)	0.2771 (6)	0.066 (3)	
H9	0.8164	0.7788	0.2792	0.079*	
C10	0.7153 (13)	0.7180 (15)	0.3276 (6)	0.079 (4)	
H10	0.7582	0.7892	0.3644	0.094*	
C11	0.6171 (13)	0.6182 (15)	0.3234 (7)	0.073 (3)	
H11	0.5943	0.6208	0.3579	0.087*	
C12	0.5536 (11)	0.5173 (14)	0.2705 (6)	0.067 (3)	
H12	0.4874	0.4504	0.2687	0.081*	
C13	0.5842 (11)	0.5110 (13)	0.2195 (6)	0.065 (3)	
H13	0.5369	0.4424	0.1823	0.078*	
C14	0.8391 (11)	0.2945 (12)	0.2415 (5)	0.054 (3)	
C15	0.9571 (13)	0.3811 (16)	0.2788 (7)	0.077 (3)	
H15	0.9813	0.4584	0.2567	0.092*	
C16	1.0364 (12)	0.3537 (17)	0.3468 (8)	0.079 (4)	
H16	1.1129	0.4146	0.3721	0.095*	
C17	1.0011 (17)	0.233 (2)	0.3775 (6)	0.099 (5)	
H17	1.0568	0.2110	0.4237	0.118*	
C18	0.8876 (15)	0.1465 (16)	0.3424 (6)	0.088 (4)	
H18	0.8648	0.0666	0.3641	0.106*	
C19	0.8083 (12)	0.1796 (13)	0.2748 (6)	0.066 (3)	
H19	0.7300	0.1213	0.2503	0.079*	

### Atomic displacement parameters (Å^2^)

**Table:**

	*U*^11^	*U*^22^	*U*^33^	*U*^12^	*U*^13^	*U*^23^
Cu	0.0701 (13)	0.0335 (10)	0.0702 (13)	0.0039 (9)	0.0374 (10)	0.0044 (8)
Cl1	0.088 (2)	0.0415 (16)	0.0687 (18)	0.0019 (14)	0.0247 (17)	−0.0021 (13)
Cl2	0.128 (3)	0.0549 (18)	0.082 (2)	0.0388 (18)	0.064 (2)	0.0194 (15)
N1	0.074 (6)	0.047 (5)	0.056 (5)	0.011 (4)	0.040 (5)	0.007 (4)
N2	0.101 (7)	0.032 (5)	0.069 (6)	−0.004 (5)	0.053 (6)	−0.001 (4)
N3	0.077 (6)	0.044 (5)	0.053 (5)	−0.005 (5)	0.034 (5)	0.001 (4)
C1	0.055 (7)	0.037 (6)	0.074 (8)	−0.001 (5)	0.033 (6)	0.004 (5)
C2	0.074 (8)	0.053 (7)	0.063 (8)	−0.006 (6)	0.040 (7)	−0.003 (6)
C3	0.062 (8)	0.065 (8)	0.090 (9)	0.016 (6)	0.046 (7)	0.006 (6)
C4	0.103 (11)	0.054 (8)	0.114 (11)	0.028 (7)	0.065 (10)	0.005 (7)
C5	0.096 (10)	0.082 (9)	0.100 (11)	0.004 (9)	0.067 (9)	−0.011 (8)
C6	0.095 (11)	0.077 (9)	0.078 (9)	−0.003 (8)	0.036 (8)	−0.023 (7)
C7	0.079 (8)	0.065 (8)	0.087 (9)	0.005 (7)	0.047 (8)	−0.006 (7)
C8	0.075 (8)	0.046 (6)	0.055 (7)	0.010 (6)	0.031 (6)	0.013 (5)
C9	0.076 (8)	0.051 (7)	0.069 (8)	−0.005 (6)	0.034 (7)	−0.006 (6)
C10	0.097 (10)	0.070 (8)	0.067 (8)	0.007 (8)	0.039 (8)	−0.011 (6)
C11	0.092 (9)	0.063 (8)	0.086 (9)	0.014 (7)	0.061 (8)	0.009 (7)
C12	0.066 (7)	0.067 (8)	0.083 (8)	0.001 (6)	0.047 (7)	−0.002 (7)
C13	0.076 (8)	0.052 (7)	0.068 (7)	−0.016 (6)	0.036 (7)	−0.002 (6)
C14	0.064 (8)	0.037 (6)	0.069 (8)	0.009 (5)	0.039 (7)	0.003 (5)
C15	0.081 (9)	0.078 (9)	0.080 (9)	0.013 (8)	0.047 (8)	0.003 (7)
C16	0.055 (8)	0.072 (9)	0.100 (11)	−0.001 (7)	0.031 (8)	−0.014 (8)
C17	0.109 (12)	0.110 (13)	0.056 (8)	0.042 (11)	0.025 (9)	−0.004 (9)
C18	0.112 (11)	0.069 (9)	0.059 (9)	−0.006 (9)	0.022 (8)	0.004 (7)
C19	0.079 (8)	0.050 (7)	0.062 (8)	0.000 (6)	0.030 (7)	0.003 (6)

### Geometric parameters (Å, °)

**Table:**

Cu—Cl2	2.263 (3)	C7—H7	0.9300
Cu—Cl2^i^	2.263 (3)	C8—C13	1.358 (14)
Cu—Cl1	2.265 (3)	C8—C9	1.400 (14)
Cu—Cl1^i^	2.265 (3)	C9—C10	1.372 (15)
N1—C1	1.324 (12)	C9—H9	0.9300
N1—C2	1.404 (12)	C10—C11	1.370 (16)
N1—H1	0.8600	C10—H10	0.9300
N2—C1	1.332 (12)	C11—C12	1.332 (15)
N2—C8	1.433 (12)	C11—H11	0.9300
N2—H2	0.8600	C12—C13	1.351 (14)
N3—C1	1.347 (12)	C12—H12	0.9300
N3—C14	1.411 (12)	C13—H13	0.9300
N3—H3	0.8600	C14—C19	1.355 (14)
C2—C7	1.363 (14)	C14—C15	1.398 (15)
C2—C3	1.379 (14)	C15—C16	1.353 (15)
C3—C4	1.373 (15)	C15—H15	0.9300
C3—H3A	0.9300	C16—C17	1.377 (19)
C4—C5	1.371 (15)	C16—H16	0.9300
C4—H4	0.9300	C17—C18	1.359 (19)
C5—C6	1.356 (16)	C17—H17	0.9300
C5—H5	0.9300	C18—C19	1.356 (14)
C6—C7	1.376 (15)	C18—H18	0.9300
C6—H6	0.9300	C19—H19	0.9300
			
Cl2—Cu—Cl2^i^	180.00 (15)	C13—C8—C9	119.2 (10)
Cl2—Cu—Cl1	88.65 (10)	C13—C8—N2	122.2 (10)
Cl2^i^—Cu—Cl1	91.35 (10)	C9—C8—N2	118.5 (10)
Cl2—Cu—Cl1^i^	91.35 (10)	C10—C9—C8	119.1 (11)
Cl2^i^—Cu—Cl1^i^	88.65 (10)	C10—C9—H9	120.4
Cl1—Cu—Cl1^i^	180.00 (16)	C8—C9—H9	120.4
C1—N1—C2	127.0 (9)	C11—C10—C9	119.3 (12)
C1—N1—H1	116.5	C11—C10—H10	120.3
C2—N1—H1	116.5	C9—C10—H10	120.3
C1—N2—C8	126.2 (9)	C12—C11—C10	120.9 (11)
C1—N2—H2	116.9	C12—C11—H11	119.5
C8—N2—H2	116.9	C10—C11—H11	119.5
C1—N3—C14	127.5 (9)	C11—C12—C13	120.9 (11)
C1—N3—H3	116.3	C11—C12—H12	119.5
C14—N3—H3	116.3	C13—C12—H12	119.5
N1—C1—N2	119.6 (9)	C12—C13—C8	120.5 (11)
N1—C1—N3	120.5 (9)	C12—C13—H13	119.8
N2—C1—N3	119.8 (10)	C8—C13—H13	119.8
C7—C2—C3	118.6 (11)	C19—C14—C15	118.3 (11)
C7—C2—N1	118.2 (10)	C19—C14—N3	121.7 (10)
C3—C2—N1	123.2 (10)	C15—C14—N3	120.0 (10)
C4—C3—C2	120.0 (12)	C16—C15—C14	120.7 (12)
C4—C3—H3A	120.0	C16—C15—H15	119.6
C2—C3—H3A	120.0	C14—C15—H15	119.6
C5—C4—C3	120.5 (12)	C15—C16—C17	118.4 (13)
C5—C4—H4	119.8	C15—C16—H16	120.8
C3—C4—H4	119.8	C17—C16—H16	120.8
C6—C5—C4	119.7 (12)	C18—C17—C16	122.1 (13)
C6—C5—H5	120.2	C18—C17—H17	118.9
C4—C5—H5	120.2	C16—C17—H17	118.9
C5—C6—C7	119.8 (12)	C17—C18—C19	118.1 (13)
C5—C6—H6	120.1	C17—C18—H18	120.9
C7—C6—H6	120.1	C19—C18—H18	120.9
C2—C7—C6	121.3 (12)	C14—C19—C18	122.3 (12)
C2—C7—H7	119.4	C14—C19—H19	118.8
C6—C7—H7	119.4	C18—C19—H19	118.8
			
C2—N1—C1—N2	161.5 (10)	C13—C8—C9—C10	1.8 (16)
C2—N1—C1—N3	−17.6 (16)	N2—C8—C9—C10	179.7 (9)
C8—N2—C1—N1	152.7 (10)	C8—C9—C10—C11	0.1 (17)
C8—N2—C1—N3	−28.2 (16)	C9—C10—C11—C12	−0.9 (17)
C14—N3—C1—N1	146.9 (9)	C10—C11—C12—C13	−0.1 (18)
C14—N3—C1—N2	−32.2 (15)	C11—C12—C13—C8	2.1 (17)
C1—N1—C2—C7	141.5 (11)	C9—C8—C13—C12	−2.9 (16)
C1—N1—C2—C3	−39.4 (16)	N2—C8—C13—C12	179.3 (10)
C7—C2—C3—C4	2.3 (17)	C1—N3—C14—C19	141.6 (11)
N1—C2—C3—C4	−176.8 (10)	C1—N3—C14—C15	−41.1 (15)
C2—C3—C4—C5	−1.4 (19)	C19—C14—C15—C16	−1.8 (16)
C3—C4—C5—C6	1(2)	N3—C14—C15—C16	−179.2 (9)
C4—C5—C6—C7	−2(2)	C14—C15—C16—C17	2.9 (17)
C3—C2—C7—C6	−3.2 (17)	C15—C16—C17—C18	−2.4 (19)
N1—C2—C7—C6	175.9 (10)	C16—C17—C18—C19	1(2)
C5—C6—C7—C2	3.1 (19)	C15—C14—C19—C18	0.1 (16)
C1—N2—C8—C13	−41.0 (15)	N3—C14—C19—C18	177.5 (10)
C1—N2—C8—C9	141.1 (11)	C17—C18—C19—C14	0.4 (18)

Symmetry codes: (i) −*x*+1, −*y*, −*z*.

### Hydrogen-bond geometry (Å, °)

**Table:**

*D*—H···*A*	*D*—H	H···*A*	*D*···*A*	*D*—H···*A*
N1—H1···Cl2^ii^	0.86	2.28	3.126 (8)	167
N2—H2···Cl1^iii^	0.86	2.51	3.218 (8)	140
N3—H3···Cl1	0.86	2.44	3.253 (9)	159

Symmetry codes: (ii) −*x*+1, −*y*+1, −*z*; (iii) *x*, *y*+1, *z*.
